# Histopathological investigation of complex gill disease in sea farmed Atlantic salmon

**DOI:** 10.1371/journal.pone.0222926

**Published:** 2019-10-03

**Authors:** Mona C. Gjessing, Terje Steinum, Anne Berit Olsen, Kai Inge Lie, Saraya Tavornpanich, Duncan J. Colquhoun, Anne-Gerd Gjevre

**Affiliations:** 1 Norwegian Veterinary Institute, Oslo, Norway; 2 Norwegian Veterinary Institute, Bergen, Norway; Friedrich-Loeffler-Institut Bundesforschungsinstitut fur Tiergesundheit, GERMANY

## Abstract

Various agents including *Ca*. Piscichlamydia salmonis, *Ca*. Branchiomonas cysticola, *Desmozoon lepeophtherii*, *Paramoeba perurans* and salmon gill poxvirus may be associated with complex gill disease in Atlantic salmon. Co-infections involving two or more of these agents are common and histopathological interpretation of lesions is therefore challenging. In this study, we developed a semi-quantitative scoring system for examination of histopathological gill lesions in sea-farmed Atlantic salmon suffering from gill disease. Following qPCR analysis of gills sampled for *Ca*. P. salmonis, *Ca*. B. cysticola, *D*. *lepeophtherii* and *P*. *perurans* from 22 geographically spread outbreaks, five cases representing different infectious loads and combinations of agents were chosen for histopathological scoring. Twenty-eight histological features were evaluated and potential associations between individual pathological changes and the occurrence of individual agents studied. The inter-observer agreement in interpretation of histological parameters between the three pathologists involved, was calculated to validate robustness of the scoring scheme. Seventeen histological parameters met the criteria for inter-observer agreement analysis and were included in the calculation. The three most frequent findings were identification of subepithelial leukocytes, epithelial cell hyperplasia and mucus cell hyperplasia. While few findings could be specifically related to particular agents, necrosis in hyperplastic lesions, pustules and necrosis of subepithelial cells appeared to be associated with the presence of *Ca*. B. cysticola. Further, lesion profiles clearly support the previously identified association between *P*. *perurans* and pathological changes associated with AGD. Very few pathological changes were observed in the single case in which *Ca*. P. salmonis was the dominating agent. Some lesions were only very rarely observed e.g. chloride cell necrosis, epithelial cell apoptosis, lamellar deposition of melanin and haemophagocytosis. The scoring scheme developed and applied was robust and sensitive. A less extensive scheme for routine diagnostic use is proposed.

## Introduction

While respiration may be the gill’s primary function, the gill is a physiologically diversified organ [1]. Compromised gill function has, therefore, a broad impact on the health of affected fish. Gill disease often has a multifactorial etiology and a complex histopathological manifestation. In this paper, we use the term ‘complex gill disease (CGD)’ when referring to these manifestations. CGD continues to be a significant challenge in the farming of Atlantic salmon in Norway and has also been reported in other salmon producing countries [2–4]. Gill diseases may involve both infectious and non-infectious agents [3–6]. Identification of the etiological roles of individual infectious agents is challenging [6–14] and with the exception of amoebic gill disease (AGD), there are no efficient treatments. Infectious agents associated with gill disease in salmon include *Paramoeba perurans*, the causative agent of AGD [15], *Desmozoon lepeophtherii* [10, 13, 16, 17], various species of intracellular and extracellular bacteria e.g. *Ca*. Piscichlamydia salmonis [18], *Ca*. Branchiomonas cysticola [8], *Ca*. Sygnamydia salmonis [19], *Tenacibaculum* spp. ([3, 20]) and viral agents including Atlantic salmon paramyxovirus [9] and salmon gill pox virus (SGPV) [21–23].

Hyperplasia of respiratory epithelium, inflammatory responses, degenerative changes and circulatory disturbances are common findings in diseased gills. Hence, terms such as proliferative gill inflammation (PGI) [12] and proliferative gill disease (PGD; also used to described a disease caused by the myxozoan parasite *Henneguya* spp. in catfish), have also been used to describe CGD [4, 24, 25]. In 2013, the Gill Health Initiative, an international network working to improve gill health in Atlantic salmon, identified a need for specific diagnostic criteria to assess gill diseases [2]. The general lack of experimental models for infectious gill diseases has hindered elucidation of possible pathognomonic changes characterizing single or multiple agent infections. While a number of histopathological scoring systems have been developed for terrestrial animals [26, 27] and attempts have been made to standardize interpretation of gill lesions in fish [28, 29], no system has yet been taken into broad use. Given the importance of gill disease in Atlantic salmon farming, both economically and in terms of animal welfare, there is an urgent need to increase our understanding of the impact of individual agents and possible synergetic effects of co-infections in gill disease.

The main aim of the present study was to establish a common understanding of CGD related histopathology by focusing on a wide range of gill lesions. Further, we wanted to extend the systematic approach proposed by Mitchell and Rodger [29] to assess the most significant histopathological gill lesions in sea-farmed Atlantic salmon.

Initially, an inter-observer agreement among three pathologists was assessed to validate the robustness of the method. Thereafter, this method was used to investigate potential associations between histopathological findings and the presence of *Ca*. P. salmonis, *Ca*. B. cysticola, *D*. *lepeophtherii*, *P*. *perurans* and SGPV in single and multiple agent field infections.

## Material and methods

### Ethical considerations

The fish used in this study were kept for commercial purposes at salmon sea farms. Gill disease was suspected and the animals were euthanized with an overdose of anaesthetics before sampling. The handling of the live fish was done in accordance with Norwegian regulations.

### Sea farms and agent detection

Gills from a total of 504 Atlantic salmon from 22 sea farms (Table 1) with suspected gill disease were qPCR tested for ‘*Ca*.’ P. salmonis [18], ‘*Ca*.’ B. cysticola [30], *D*. *lepeophtherii* [16] and *P*. *perurans* [31] as previously described. Gills from 88 fish included in the histopathological investigation (Table 1) were also analysed for SGPV [21]. Most of the material was collected during the late summer and autumn months in 2012 and 2013.

**Table 1:** Overview of geographical distribution of farms (Case ID), number of fish included from each case and agent load. Abbreviations: ND = not detected, NA = not analyzed. Samples from cases in bold were included in the histopathological investigation; ^12^ = twelve and ^6^ = six fish were tested for salmon gill poxvirus.

Part of Norway	Case ID	Number of fish	% positive fish for different microorganisms (median RT qPCR Ct-value for positives ± MAD)				
			*‘Ca*. Branchiomonas cysticola’	*Desmozoon lepeophtherii*	‘*Ca*. Piscichlamydia salmonis’	*Paramoeba perurans*	Salmon gill poxvirus
Southern	**1**	**10**	**60% (29.9±1.6)**	**100% (30.9±1.9)**	**100% (22.3±1.6)**	**ND**	**10% (29.7)**
	2	17	100% (25.4±1.4)	94% (24.7±1.4)	53% (30.4±0.4)	ND	NA
	**3**	**30**	**100% (23.8±2.8)**	**100% (24.9±1.4)**	**ND**	**100% (25.2±2.2)**	**17% (33.4±3.3)**
	4	10	60% (31.0±3.0)	100% (24.1±0.9)	80% (29.3±1.1)	70% (30.8±1.9)	NA
	5	10	100% (28.7±1.0)	100% (25.9±1.8)	50% (30.6±1.4)	80% (28.5±2.4)	NA
	6	30	100% (22.9±1.7)	100% (30.9±2.1)	17% (33.4±4.2)	ND	NA
	7	30	93% (28.4±2.7)	97% (29.0±2.6)	90% (27.2±2.7)	ND	NA
	8	30	100% (21.7±2.3)	100% (24.4±1.4)	40% (31.3±2.2)	100% (24.9±1.2)	NA
Mid	9	10	100% (23.7±3.5)	90% (26.4±1.5)	70% (27.3±1.1)	ND	NA
	10	30	100% (26.0±2.7)	93% (27.0±3.2)	93% (28.6±1.6)	ND	NA
	**11**	**16**	**100% (20.3±1.3)**	**100% (22.4±3.4)**	**ND**	**ND**	**42% (27.1±0.6)**^**12**^
	12	30	97% (25.4±1.5)	97% (26.9±1.5)	ND	ND	NA
	**13**	**15**	**100% (18.8±1.3)**	**100% (24.8±1.5)**	**ND**	**20% (30.2±0.9)**	**67% (29.3±3.1)**^**6**^
	14	30	100% (27.8±1.4)	80% (30.7±2.0)	3% (37.5)	ND	NA
	15	29	100% (23.5±4.5)	100% (25.3±3.0)	30% (30.0±2.3)	ND	NA
	16	30	100% (17.4±2.1)	17% (37.0±0.8)	7% (30.4±3.0)	3% (37.3)	NA
North	17	17	82% (25.5±1.7)	59% (30.3±1.4)	100% (26.2±2.1)	ND	NA
	18	10	100% (26.8±1.2)	60% (29.4±2.2)	50% (29.4±1.4)	ND	NA
	19	44	100% (22.4±2.3)	ND	2% (37.1)	2% (30.2)	NA
	20	16	100% (23.0±1.7)	ND	13% (32.9±0.5)	ND	NA
	21	30	80% (30.9±1.4)	33% (30.7±1.5)	67% (29.7±1.7)	ND	NA
	**22**	**30**	**100% (24.7±2.6)**	**ND**	**ND**	**ND**	**27% (33.7±0.6)**
	All farms	504	91% (24.7±3.2)	71% (26.6±2.7)	32.8% (28.5±2.5)	15% (25.9±2.4)	**26% (34.0±2.7)**

### Selection of cases

Samples from five (indicated in bold text in Table 1*)* of the 22 geographically spread farms (five ‘cases’) with different agent profiles, as determined by qPCR, were chosen for histopathological investigation. In addition, gills of 12 fish experimentally exposed to *P*. *perurans* were evaluated. Two of the selected cases were dominated by ‘*Ca*.’ P. salmonis and ‘*Ca*.’ B. cysticola respectively (Table 1, farms 1 and 22), two were co-infected with ‘*Ca*.’ B. cysticola and *D*. *lepeophtherii* (Table 1, farms 11 and 13) and in one case ‘*Ca*.’ B. cysticola, *D*. *lepeophtherii* and *P*. *perurans* were present in all fish studied (Table 1, farm 3).

### Preparation of samples

For histopathological evaluation the second left gill arch from each fish was fixed in 10% neutral phosphate buffered formalin, embedded in paraffin and 3 μm thick sections were stained with haematoxylin and eosin (H&E) according to standard protocols. In addition, a subset of sections were stained to detect chloride cells as previously described [21].

### Inter-observer agreement

The level of agreement among three pathologists in scoring of severity for each histopathological lesion type was assessed by estimation of Intra-class Correlation Coefficient (ICC) [32] on the field samples. ICC estimates and 95% confident intervals were calculated using R software [33], package “irr” based on a fully crossed design, two-way random effect model, type of agreement between raters.

The calculation implied that features scored 0 (not present) by all three pathologists were excluded. ICC ranges from– 1 to 1. In this study, we used Cicchetti’s cut-off to categorise ICC values of < 0.40 as poor agreement, 0.40–0.59 as fair agreement, 0.60–0.74 as good agreement, and 0.75 and 1.00 as excellent agreement [34].

### Histopathological examination

**Fig 1:**
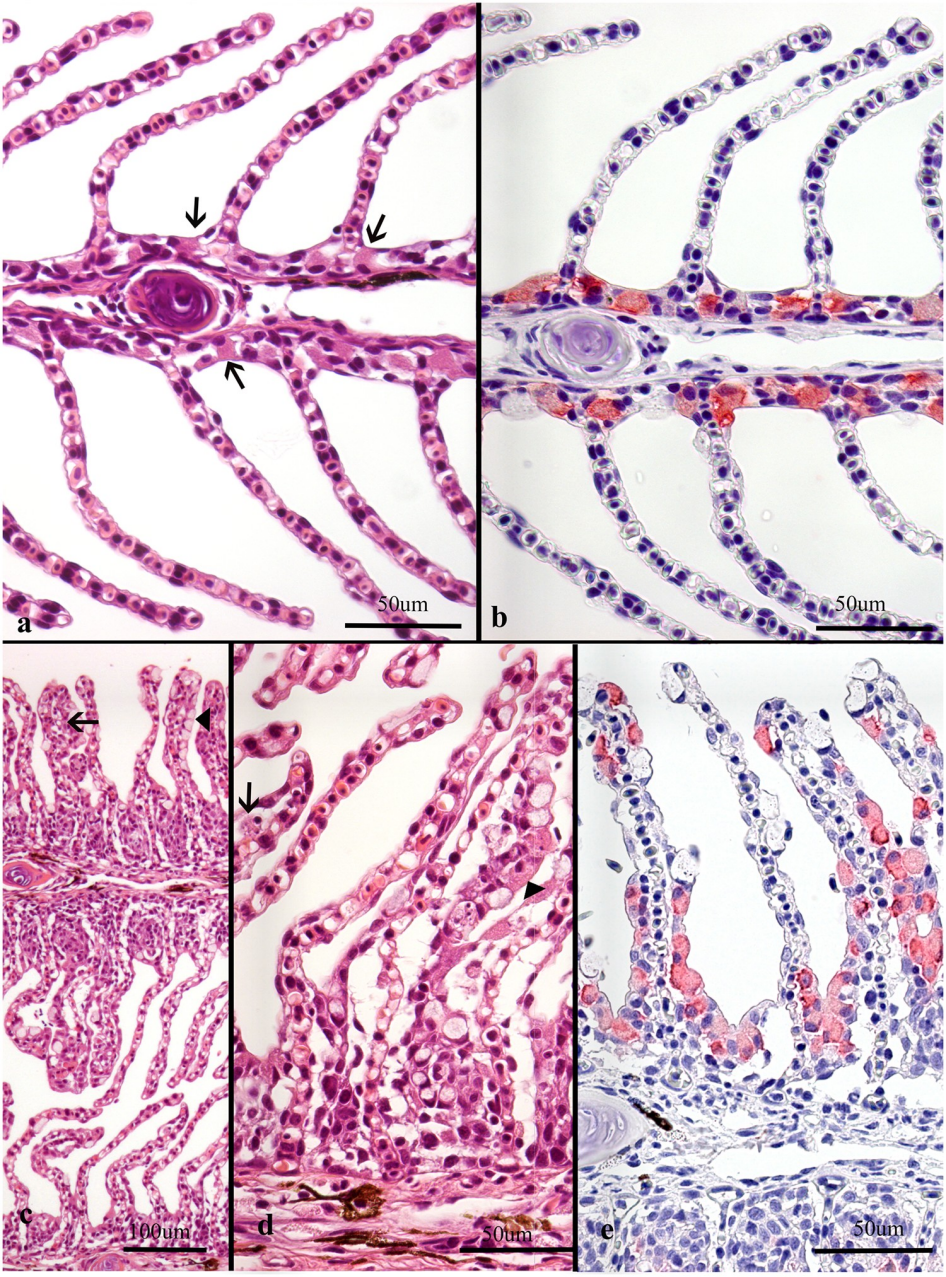
Histological sections of gills from Atlantic salmon. Note that plane of section is consistently on the efferent side of the gills in all images as judged by the small amount of filament cartilage. Panel b and e are sections stained by IHC for chloride cells (red). (a-b) Normal gill with thin lamellae. (a) Chloride cells (arrows) are seen in the interlamellar space, more clearly seen in (b). Note that the chloride cells are not seen along the lamellae. (c-d) Gill from farm 22, dominated by infection by *Ca*. Branchiomonas cysticola. (c) Lamellae with little adhesions, although thicker than normal due to mucus cell hyperplasia (arrowhead) scored 2, some chloride cell hyperplasia, scored 3 and subepithelial leukocytes (arrow), scored 6. Some epithelial cell hyperplasia is also seen basally in the interlamellar space, scored 3. (d) Magnification of same gill as seen in c with subepithelial leukocytes and necrotic cells (arrow) and chloride cell hyperplasia (arrowhead). (e) Same gill as in d, more clearly demonstrating chloride cell hyperplasia.

**Fig 2:**
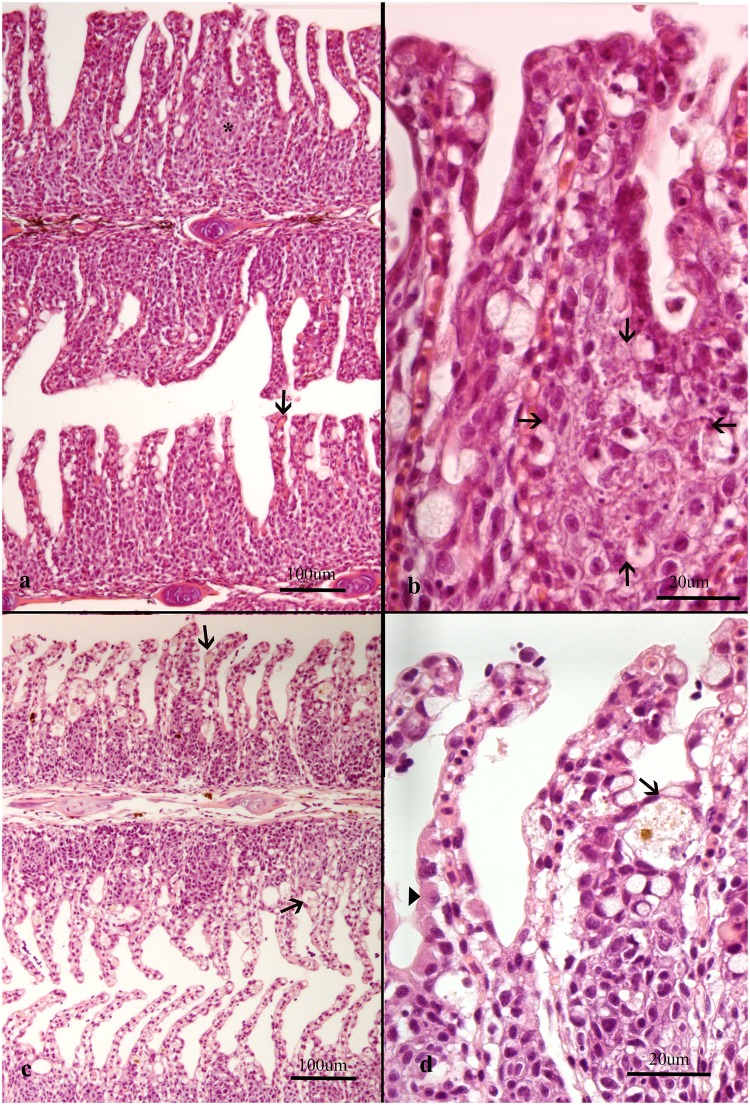
Histological sections of gills from Atlantic salmon. Note that plane of section is consistently on the efferent side of the gills in all images as judged by the small amount of filament cartilage. (a-b) Gills from farm 13 were dominated by *Desmozoon lepeoptherii* and *Ca*. Branchiomonas cysticola. (a) Epithelial cell hyperplasia scored 6, some thrombosis (arrowhead) and necrosis in hyperplastic lesions (arrow) scored 5, an area enlarged in (b) (arrows). (c-d) Gills from farm 3 were dominated by *Ca*. Branchiomonas cysticola and *Desmozoon lepeoptherii*. (c) Epithelial cell hyperplasia scored 4, ballooning degenerative cells containing pigmented material (arrows), scored 6. (d) Enlarged area from c with chloride cell hyperplasia (arrowhead) and ballooning degenerative cells containing pigmented material (arrow).

**Fig 3:**
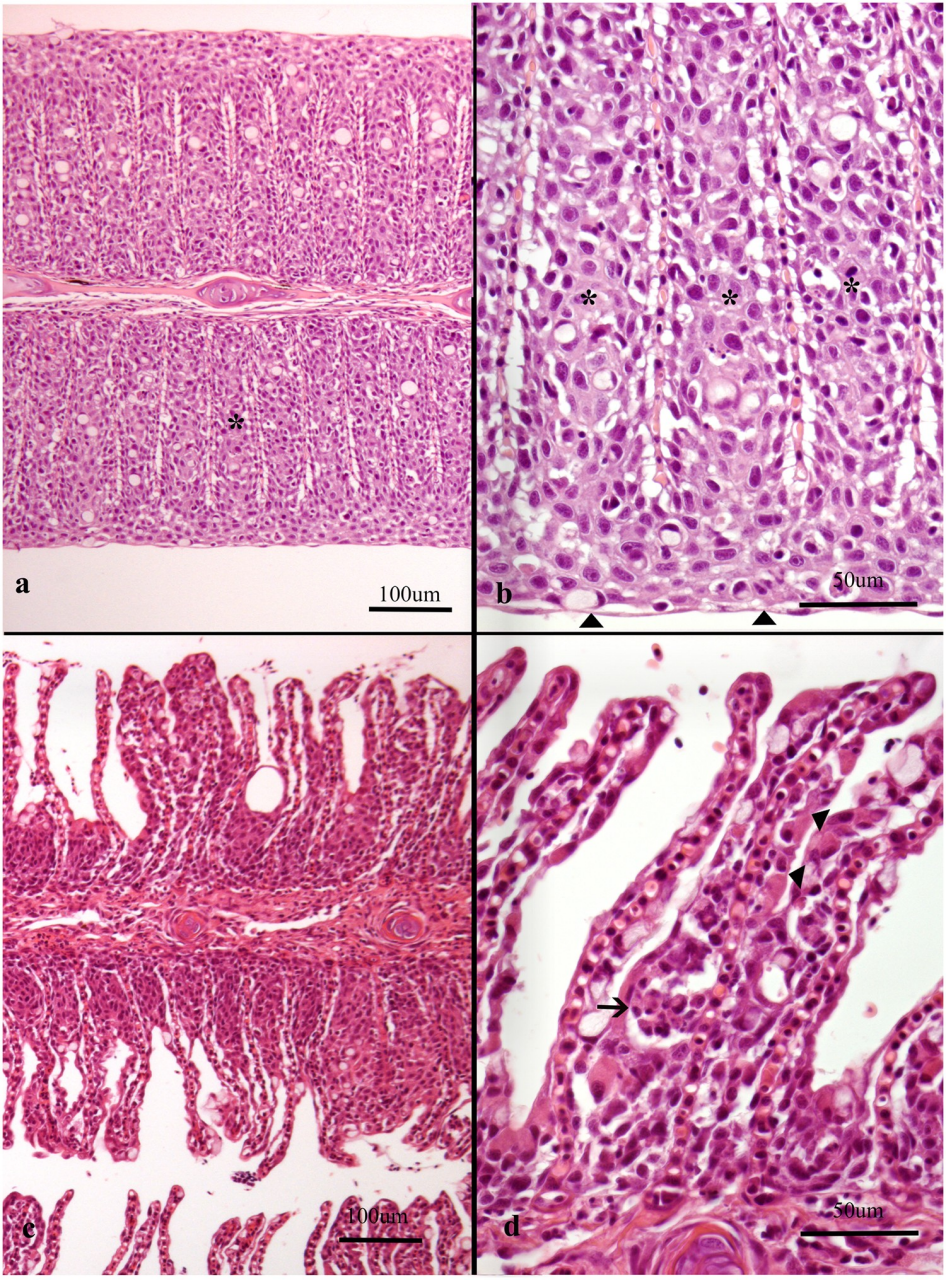
Histological sections of gills from Atlantic salmon. Note that plane of section is consistently on the efferent side of the gills in all images as judged by the small amount of filament cartilage. (a-b) Gills from farm 3 were dominated by *Paramoeba perurans* in addition to *Ca*. Branchiomonas cysticola and *Desmozoon lepeoptherii*. (a) Note extensive epithelial cell hyperplasia (asterisk) (scored 7) causing total obstruction of respiratory surface (b) extensive epithelial cell hyperplasia (asterisk) and metaplasia (arrowheads), scored 5. Note apparently unaffected lamellar vessels. (c-d) Gills from farm 22 were dominated by *Ca*. Branchiomonas cysticola. (c) note thickened lamellae caused by epithelial cell hyperplasia, scored 5 and some mucus cell hyperplasia, scored 3 and subepithelial leukocytes, scored 5. (d) subepithelial inflammatory cells and dead cells (arrow) and chloride cell hyperplasia (arrowheads), scored 3.

**Fig 4:**
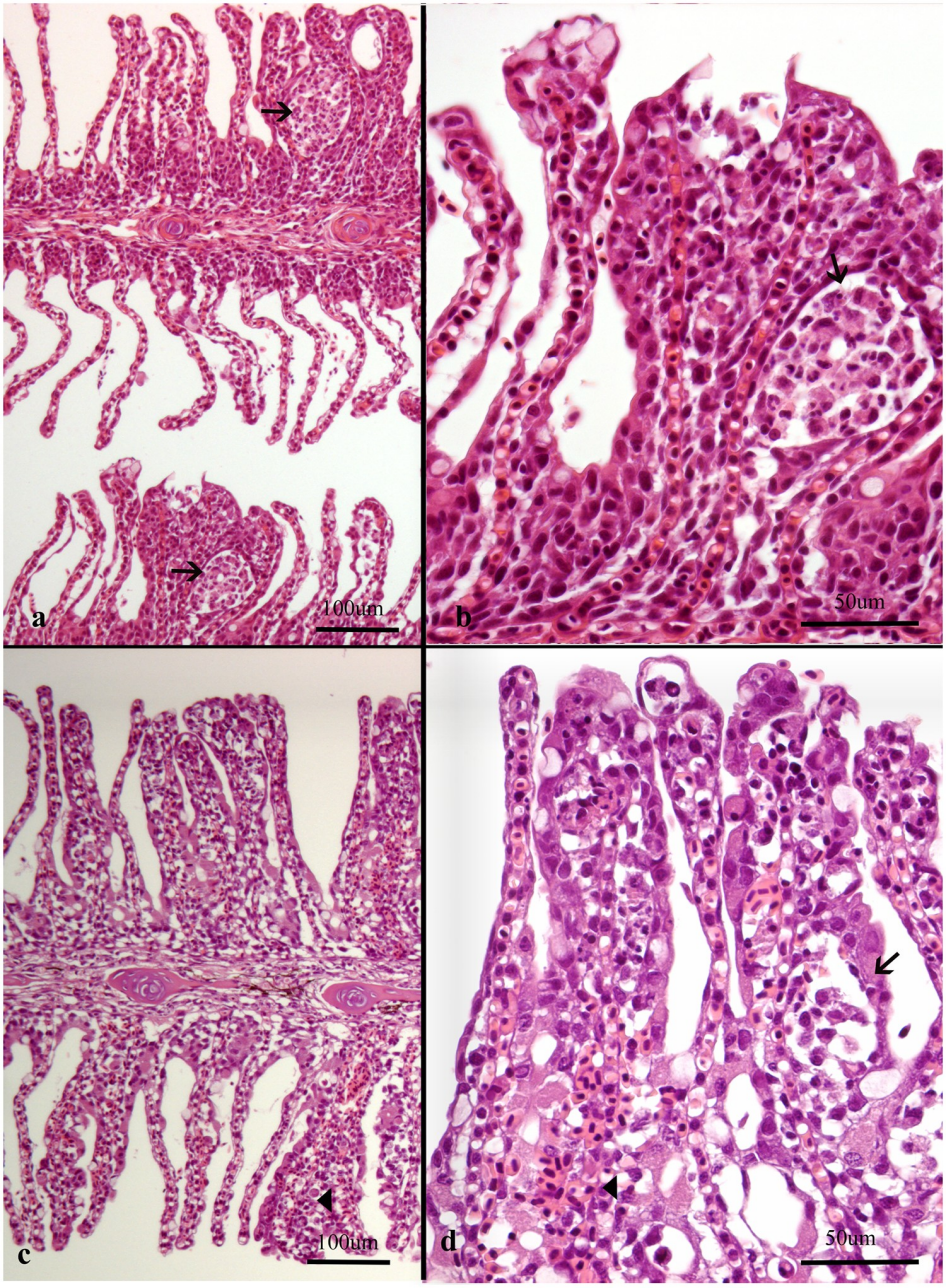
Histological sections of gills from Atlantic salmon. Note that plane of section is consistently on the efferent side of the gills in all images as judged by the small amount of filament cartilage. (a-b) Gills from farm 22 were dominated by *Ca*. Branchiomonas cysticola. (a) Epithelial cell hyperplasia and pustules (arrows), scored 5 (b) large pustule (arrow) in proliferative lesion (c-d) Gills from farm 3 were dominated by *Ca*. Branchiomonas cysticola and *Desmozoon lepeoptherii*. Haemorrhages (arrowhead), scored 4. (d) subepithelial leukocytes (arrow), scored 5.

**Fig 5:**
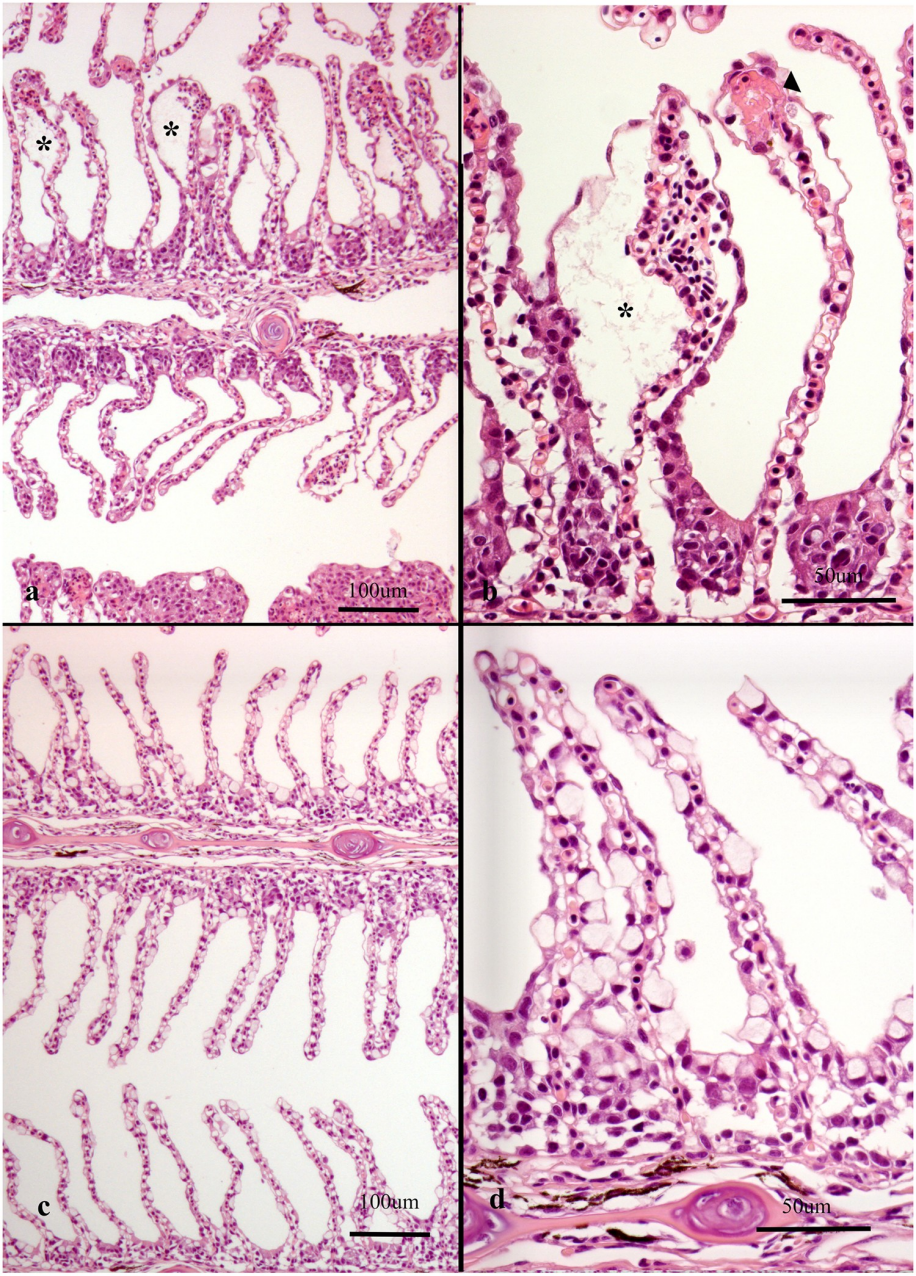
Histological sections of gills from Atlantic salmon. Note that plane of section is consistently on the efferent side of the gills in all images as judged by the small amount of filament cartilage. (a-b) Gills from farm 3 were dominated by *Ca*. Branchiomonas cysticola and *Desmozoon lepeoptherii*. (a) some lamellar adhesion and subepithelial oedema (asterisk), scored 2, (b) manifestation of subepithelial oedema (asterisk) and thrombosis (arrowhead). (c-d) mucus cell hyperplasia scored 4.

A broad panel of 28 different types of histopathological change previously described in salmon gills were selected for examination (Table 2). The gill sections were examined (blinded investigation) by three pathologists. Sections displaying artifacts e.g. compression of tissues during sampling, folding of cut tissues or autolysis due to improper fixation, were rejected. The plane of section was also taken into account. As chloride cells are present in greater numbers on the afferent side of the lamellae [35] the degree of hyperplasia of these cells was assessed on the efferent side. Lesions were assessed both qualitatively (presence or absence), and semi–quantitatively with a score between 0 and 10, where 1 indicated very mild and 10 most severe change. The lesions were categorized as degenerative, inflammatory, circulatory or adaptive (Table 2, Figs 1–5) [5, 6, 9, 10, 13, 21, 36]. In addition, sloughing of epithelial cells, marginal lamellar adhesions and histologically visible microorganisms were registered. Although the term “pustule” traditionally refers to a purulent lesion in the skin, we have chosen to use this term in the context of gill diseases when we see aggregations of leukocytes as shown in Fig 4b.

**Table 2:** Overview of histopathological features included in the scoring system, literature references and references to figures demonstrating the lesions.

Feature ID	Degenerative changes	References and figures
1	Epithelial cell apoptosis	[21–23]
2	Epithelial cell necrosis	[5]
3	Subepithelial cell necrosis	[12] Fig 1
4	Necrosis in hyperplastic lesions	[9, 12] Fig 2
5	Ballooning in degenerative cells containing pigmented material	Fig 2
6	Chloride cell necrosis	[21]
7	Presence of micro-vesicles	[10, 13] Fig 3
Feature ID	Inflammatory changes	References and figures
8	Subepithelial leukocytes	[9, 12], Fig 3
9	Pustules	Fig 4
10	Inflammatory cells in filament sinusoid	[9, 14]
11	Inflammatory cells in filament	-
Feature ID	Circulatory changes	References and figures
12	Haemorrhage	[9], Fig 4
13	Telangiectasiae	-
14	Haemophagocytosis	-
15	Subepithelial oedema	[9], Fig 5
16	Thrombosis in lamellar vessels	[9], Fig 5
Feature ID	Adaptive changes	References and figures
17	Epithelial cell hypertrophy	-
18	Epithelial cell hyperplasia	Figs 2–4
19	Chloride cell hyperplasia	Fig 3
20	Mucus cell hyperplasia	Fig 5
21	Epithelial metaplasia	Fig 3
22	Melanin deposition in lamellae	-
Feature ID	Other findings, including agents	References and figures
23	Epiteliocysts	[8, 12, 23]
24	Amoebae (*Paramoeba perurans*-like)	[11, 14, 23]
25	Costia (*Ichtyobodo* sp.-like)	[23]
26	*Trichodina* sp.-like	-
27	Sloughing of epithelial cells	-
28	Marginal lamellar adhesions	-

### Histopathological characteristics at case level

In order to highlight the most dominant lesions in each case, the distribution of individual lesion scores from all pathologists were displayed using a box plot. Only lesions scored above zero by all pathologists in more than four samples are included in the profile.

## Results

### Agent detection

With one exception, ‘*Ca*.’ B. cysticola was detected at a high prevalence in all 22 farms (Table 1). *D*. *lepeophtherii* was detected at high prevalence in all farms in Southern and mid-Norway and at lower prevalence in Northern Norway. ‘*Ca*.’ P. salmonis and *P*. *perurans* were the least prevalent agents identified in the present study. The level and prevalence of SGPV in the five farms tested was very low. In four farms in Southern Norway, AGD was suspected by fish health services and the suspicion was further strengthened following PCR demonstration of a high prevalence of *P*. *perurans*. Other agents tested by pcr in this study, were also present in all four farms.

### Inter-observer agreement

Seventeen of the 28 gill histopathological parameters met the criteria for inclusion in the analysis (Table 3, Figs 1–5). “Ballooning, degenerative cells containing pigmented material” (Fig 2), “inflammatory cells in filament sinusoid”, “epithelial cell hyperplasia” (Figs 1–3), “epitheliocysts”, “amoebae” and “marginal lamellar adhesion” showed excellent agreement.

**Table 3:** Overview of inter-observer scoring agreement among the three pathologists. The level of agreement for each histopathological parameter was assessed by estimation of Intra-class Correlation Coefficient (ICC = ranges from– 1 to 1). ICC values of < 0.40 indicates poor agreement, 0.40–0.59 fair agreement, 0.60–0.74 good agreement, and 0.75 and 1.00 as excellent agreement. CI: confidence interval.

Lesion ID	Degenerative changes	No. of samples scored 0 by all pathologists	No. of samples scored over 0 by all pathologists	ICC (95% CI)
5	Ballooning degenerative cells containing pigmented material	52	5	0.89 (0.51–0.99)
4	Necrosis in hyperplastic lesions	43	16	0.69 (0.25–0.88)
3	Subepithelial cell necrosis	18	28	0.46 (0.02–0.73)
6	Chloride cell necrosis	74	0	NA
7	Microvesicles	55	0	NA
1	Epithelial cell apoptosis	70	0	NA
2	Epithelial cell necrosis	50	0	NA
Lesion ID	Inflammatory changes			
10	Inflammatory cells in filament sinusoid	22	10	0.81 (0.45–0.95)
8	Subepithelial leukocytes	2	55	0.68 (0.39–0.83)
9	Pustules	43	18	0.47 (-0.14–0.78)
11	Inflammatory cells in filament	22	5	0.35 (-0.19–0.88)
Lesion ID	Circulatory changes			
13	Telangiectasia	38	16	0.74 (0.42–0.90)
12	Haemorrhages	36	18	0.67 (0.25–0.87)
16	Thrombosis in lamellar vessels	32	13	0.34 (-0.33–0.75)
14	Haemophagocytosis	45	0	NA
15	Subepithelial oedema	64	3	NA
Lesion ID	Adaptive changes			
18	Epithelial cell hyperplasia	6	52	0.91 (0.84–0.94)
19	Chloride cell hyperplasia	22	11	0.47 (-0.21–0.83)
20	Mucus cell hyperplasia	5	41	0.59 (0.27–0.78)
21	Epithelial metaplasia	38	24	0.43 (-0.14–0.74)
17	Epithelial cell hypertrophy	51	4	NA
22	Melanin in lamellae	57	0	NA
Lesion ID	Other findings, including agents			
23	Epitheliocysts	31	14	0.83 (0.57–0.94)
24	Amoebae (*Paramoeba perurans*-like)	62	13	0.79 (0.45–0.93)
28	Marginal lamellar adhesion	21	34	0.78 (0.50–0.90)
26	*Trichodina* sp.-like	64	4	NA
25	Costia (*Ichtyobodo* sp.-like)	73	2	NA
27	Sloughing of epithelial cells	56	0	NA

“Necrosis in hyperplastic lesions” (Fig 2), “subepithelial leukocytes” (Figs 3 and 4), “telangiectasia” and “hemorrhages” (Figs 4 and 5) showed good agreement. “subepithelial necrosis” (Fig 1), “pustules” (Fig 4), “chloride cell hyperplasia” (Figs 1 and 2), “mucus cell hyperplasia” (Fig 5) and ”epithelial metaplasia” (Fig 3) showed fair agreement. “Inflammatory cells within filament” and “thrombosis in lamellar vessels” (Fig 5) showed poor agreement.

Eleven of the 28 histopathological characteristics could not be included in the ICC analysis, as less than five samples for each characteristic scored above zero by all pathologists.

### Histopathological findings

Of the 113 fish selected for histopathological examination, 6 were rejected due to artifact or autolysis. The three most frequent histopathological characteristics observed were the inflammatory change “subepithelial leucocytes” and the adaptive changes “epithelial cell hyperplasia” and “mucus cell hyperplasia”. Only two, six and five sections respectively were scored zero by all pathologists for these lesions (Table 3). Seven histopathological parameters were scored as zero by all three pathologists. These were “necrosis of chloride cells”,”microvesicles”, “apoptosis of epithelial cells”, “necrosis of epithelial cells”, “haemophagocytosis”, “melanin in lamellae” and “sloughing of epithelial cells”. In addition, “epithelial cell hypertrophy”, “subepithelial oedema” “*Trichodina* sp.” and “costia” were rarely observed (Table 3).

### Histopathological characteristics at the case level

**Fig 6:**
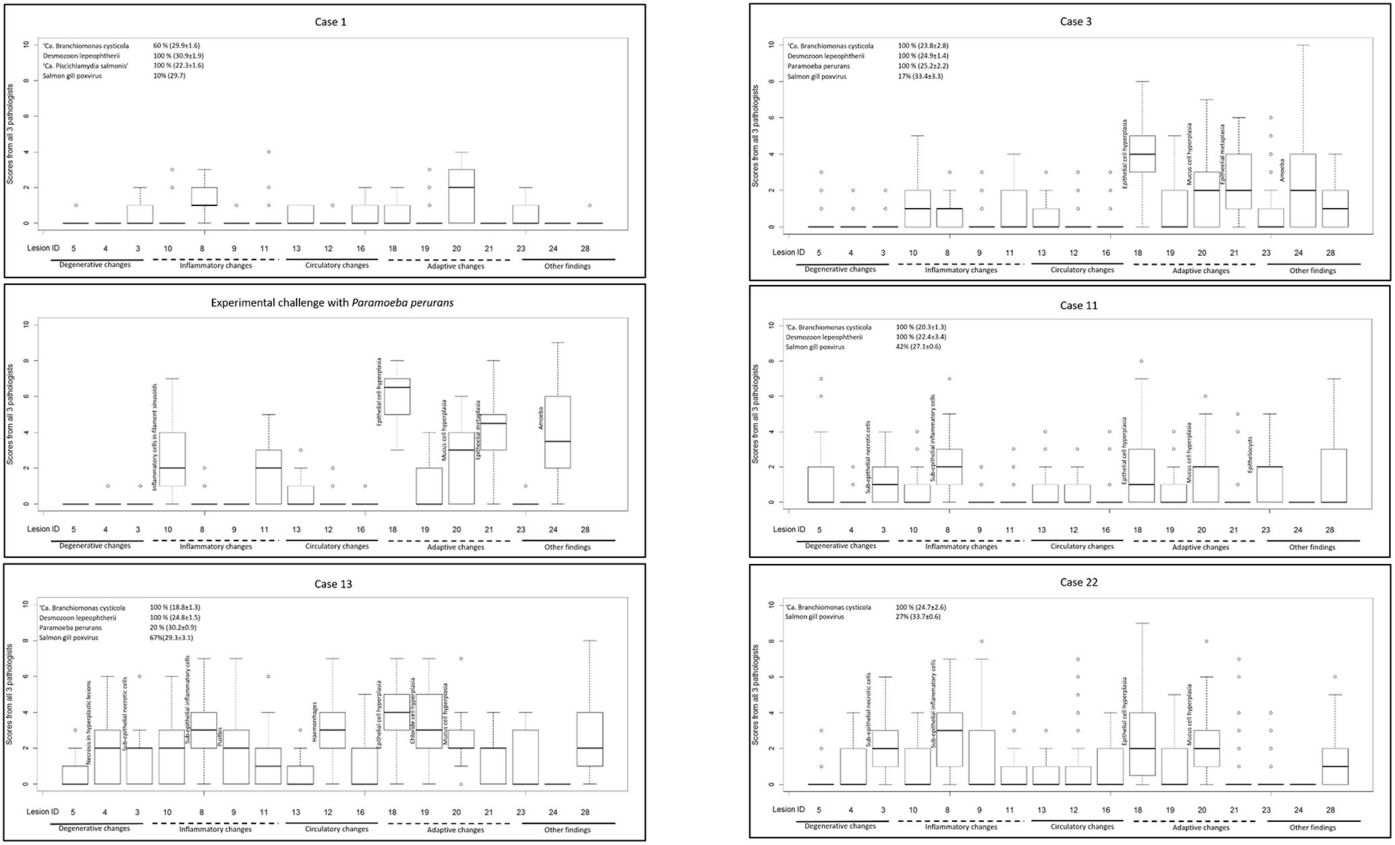
Presentation of the degree of histological lesions from the cases included in the scoring. Of the 28 lesions scored, 17 met the criteria for the Intra-class Correlation Coefficient analysis. The box in the left upper corner represents percentage positive fish for different microorganisms (median RT qPCR Ct-value for positives ± MAD). The lesion ID are explained in Table 2.

Some degree of adaptive and inflammatory changes were observed in all cases (Fig 6), whereas circulatory and degenerative changes were absent or nearly absent in some cases.

In sections from farm 1 in which *Ca*. P. salmonis was the dominating agent, very few histological changes were observed (Fig 6).

In case 3 and the experimental challenge, both involving heavy infestation of *P*. *perurans*, a very similar lesion profile was observed. Here, lesions were mainly adaptive including epithelial cell metaplasia (squamous epithelial cells were common (Fig 3)), epithelial cell hyperplasia, mucus cell hyperplasia and the notable presence of amoebae. Degenerative and circulatory changes were nearly absent and the slight degree of inflammation observed consisted of low numbers of inflammatory cells within filaments and filament sinusoids (Fig 6). Larger numbers of inflammatory cells were observed in filament sinusoids of experimentally infected AGD fish, compared to the field case.

In cases 11, 13 and 22, more complex histopathological manifestations were seen. Sections from farm 22, where with the exception of low level SGPV infection in some fish, *Ca*. B. cysticola was the only agent detected, were dominated by degenerative changes. These changes consisted of subepithelial necrotic cells, inflammatory changes consisting of subepithelial leukocytes and adaptive hyperplastic changes involving epithelial cells and mucus cells (Fig 6). Few circulatory changes were seen.

In case 11 where *Ca*. B. cysticola and *D*. *lepeoptherii* were the most abundant agents, a similar lesion profile as in case 22 was seen, but generally less pronounced (Fig 6).

Gills from case 13, with high total load and diversity of pathogens, had the most pronounced lesions. Hyperplastic lesions were dominated by degenerative subepithelial necrosis and necrosis. Inflammatory changes consisting of subepithelial leukocytes and pustules, circulatory disturbances consisting of haemorrhages and adaptive changes consisting of epithelial- mucus- and chloride cell hyperplasia were also observed. (Fig 6).

*Ca*. B. cysticola was detected by qPCR in all gills examined from all three farms involving complex infections (n = 64) (Table 1), but epitheliocysts (inclusions) were observed (by all pathologists) in only 14 gill samples (29%) (Table 3).

## Discussion

Histopathological analysis is crucial for description and understanding of a disease, but it is a subjective analytical method. Consistent assessment is therefore important to ensure the uniformity of results generated. In this study a standardized scoring form was developed including 28 histological features with a scoring scale from 0–10. An inter-observer agreement between the pathologists was determined by calculation of ICC in order to validate the robustness and reliability of the scoring system. The histological samples evaluated comprised gills from five field cases of gill disease in Atlantic salmon involving single and multiple agent infections, and from an experimental *P*. *perurans* infection. The range and complexity of histological lesions associated with gill disease in sea-farmed Atlantic salmon in Norway were clearly demonstrated.

The inclusion of the cases in the study was based on suspected gill disease as judged by field fish health personnel. A full health history of the fish groups was, however, not available and that other infectious agents or environmental conditions may have influenced the health of the studied gills cannot be discounted. Further, samples were collected only once from each farm, following identification of clinical disease, which does not give information about early stage changes and/or probable primary cause. These factors must be taken into consideration on evaluation of potential associations between particular histopathological findings and the presence of selected agents in single and multiple agent field infections.

### The established histopathological scoring method has a high inter-observer agreement

The inter-observer agreement among the three pathologists was good or excellent for 10 of the 17 parameters included in the agreement analysis, suggesting the analysis to be highly robust. This is also supported by nearly perfect agreement relating to the absence or near absence of an additional 11 lesion types considered. The broad scoring scale utilized (0 to 10) allowed a high degree of differentiation of lesion severity. However, as precise categorization on a large material is demanding, the inter-observer agreement, as well as repeatability, would most probably be maximized if a narrower scale was used [26]. Excellent inter-observer agreement was identified for conspicuous lesion types such as “ballooning degenerative cells containing pigmented material”, “inflammatory cells in filament sinusoid”, “epithelial cell hyperplasia”, “epitheliocysts”, “amoebae” and “marginal lamellar adhesion”. The poor inter-observer agreement regarding identification of “thrombosis in lamellar vessels” could be contributed to difference in recognition of degree of subtle lesions or that thrombosis may in some cases be confused with a hemorrhage that has been organized.

### Does *Ca*. B. cysticola infection cause necrosis and pustules?

The severe gill pathology associated with *Ca*. B. cysticola observed in this study is in contrast to conclusions drawn by Gunnarsson and colleagues [37]. They observed a recurring infection pattern of *Ca*. B. cysticola in sequential molecular screenings of gill pathogens in six salmon sea-farms located in an area with a high risk of developing gill disease [37]. Athough high levels of *Ca*. B. cysticola were detected, Gunnarsson *et al*. concluded that *Ca*. B. cysticola could not be directly associated with gill disease.

However, the complex histopathological manifestation observed in gills infected with both *Ca*. B. cysticola and *D*. *lepeophtherii*, in the present study corresponds well with previous studies [12, 30]. In the case where *Ca*. B. cysticola was the only agent detected, the histopathological changes observed were also complex. However, in common with previous reports [8, 12, 30] our findings suggest that necrosis in hyperplastic lesions, pustules and necrosis of subepithelial cells, appear to be specifically associated with this agent even when visible epitheliocysts are not prevalent. Such inflammatory changes, consistent with bacterial infections, are very different to those observed in *Ca*. P. salmonis infection where the lack of obvious host response was striking, particularly in light of the inflammatory response generally generated by members of the Chlamydiales pathogenic to warm-blooded animals [38, 39].

### *Ca*. P. salmonis has seemingly little impact on gill health

In the case where *Ca*. P. salmonis was the dominating agent (case 1), few histological changes were seen. Although an overall moderately positive association between *Ca*. P. salmonis and proliferative gill disease was reported by Steinum and colleagues [7] they also found several gills infected with *Ca*. P. salmonis with little pathology. This observation supports other reports suggesting that *Ca*. P. salmonis has only a minor impact on gill health [37].

### Are degenerative cells containing pigmented material a sign of *D*. *lepeophtherii* infection?

There are reports of the presence of *D*. *lepeophtherii* in gills without any obvious associated pathological findings [12]. In our study however, ballooning degenerative cells containing pigmented material were only seen in association with *D*. *lepeophtherii* infections. Similar changes have been reported by others in salmon gills severely infected with *D*. *lepeophtherii* [10, 13] suggesting an association of *D*. *lepeophtherii* to ballooning degenerative cells containing pigmented material, at least at certain stages of the infection.

### Epithelial metaplasia is highly associated to *P*. *perurans* infection

The lesion profile for the field case in which *P*. *perurans* was found in all gills and the experimental *P*. *perurans* infection confirmed the pathology of amoebic gill disease as previously described [40, 41]. The lesions are mainly adaptive and our observation of the association between epithelial metaplasia and AGD gill pathology in our material is worthy of note. Epithelial metaplasia is an adaptation that replaces one type of epithelium with another that is more likely to survive the given circumstances and in the present case, *P*. *perurans* seems to have triggered the metaplasia. Although all gills in the AGD field case were also infected with moderate levels of both Ca. *B*. *cysticola* and *D*. *lepeophterii*, the associated findings were few. Thus, the finding of typical AGD histopathology, at an advanced stage, as in our case, does not exclude simultaneous infection with Ca. *B*. *cysticola* and *D*. *lepeophterii*.

### Rarely observed lesions

Epithelial cell apoptosis is a common finding in gills of salmon suffering from SGPV disease. As far as we are aware, this type of lesion has not been reported in gills where SGPV is not present or present in low levels. Therefore, the very low levels of SGPV in the studied material is in agreement with the absence of apoptotic epithelial cells. Previous studies have clearly demonstrated that SGPV may, in some cases, play an important role in complex gill disease in Atlantic salmon [36]. Low levels of SGPV in this study may indicate that it is irrelevant in this material, or that SGPV may have been involved at an earlier stage of gill disease as suggested in previous work [36]. Also, epithelial cell necrosis was hardly seen in our study. This lesion is often reported in salmon post- algal bloom exposure [42] yet our data suggest that algal blooms were not significantly involved, at least not at the time of sampling. Likewise, circulatory related findings such as haemophagocytosis and subepithelial oedema were rarely observed. A definite identification of the aetiology of subepithelial oedema often remains unresolved, but such pathological changes may appear in fish suffering from anaemia, or following exposure to water borne toxins. Haemophagocytosis is also an non-specific finding, but may be virus induced [43]. In our experience, subepithelial oedema is not a common finding in connection with complex gill disease in Atlantic salmon.

## Conclusions

The scoring method established was robust, has a high degree of sensitivity, and is therefore time consuming and more suited for research than for routine diagnostic use. However, making a less extensive scoring system by reducing the number of parameters scored and using a narrower scoring scale (e.g. 0–5); we consider that this method also could be applicable for routine diagnostic use. Experimental models are strongly needed to elucidate the pathogenesis of single and multiple agent infections of the gill and investigate possible synergetic effects of co-infections in gill disease.

## Supporting information

S1 File Data. Scores of gill sections by three pathologists for Intra-class Correlation Coefficient (ICC) analysis.
(XLSX)
